# Efficacy of Distortion Correction on Diffusion Imaging: Comparison of FSL Eddy and Eddy_Correct Using 30 and 60 Directions Diffusion Encoding

**DOI:** 10.1371/journal.pone.0112411

**Published:** 2014-11-18

**Authors:** Haruyasu Yamada, Osamu Abe, Takashi Shizukuishi, Junko Kikuta, Takahiro Shinozaki, Ko Dezawa, Akira Nagano, Masayuki Matsuda, Hiroki Haradome, Yoshiki Imamura

**Affiliations:** 1 Department of Radiology, Nihon University School of Medicine, Tokyo, Japan; 2 Department of Oral Diagnostic Sciences, Nihon University School of Dentistry, Tokyo, Japan; 3 Department of Radiological Technology, Nihon University Itabashi Hospital, Tokyo, Japan; University of Pécs Medical School, Hungary

## Abstract

Diffusion imaging is a unique noninvasive tool to detect brain white matter trajectory and integrity in vivo. However, this technique suffers from spatial distortion and signal pileup or dropout originating from local susceptibility gradients and eddy currents. Although there are several methods to mitigate these problems, most techniques can be applicable either to susceptibility or eddy-current induced distortion alone with a few exceptions. The present study compared the correction efficiency of FSL tools, “eddy_correct” and the combination of “eddy” and “topup” in terms of diffusion-derived fractional anisotropy (FA). The brain diffusion images were acquired from 10 healthy subjects using 30 and 60 directions encoding schemes based on the electrostatic repulsive forces. For the 30 directions encoding, 2 sets of diffusion images were acquired with the same parameters, except for the phase-encode blips which had opposing polarities along the anteroposterior direction. For the 60 directions encoding, non–diffusion-weighted and diffusion-weighted images were obtained with forward phase-encoding blips and non–diffusion-weighted images with the same parameter, except for the phase-encode blips, which had opposing polarities. FA images without and with distortion correction were compared in a voxel-wise manner with tract-based spatial statistics. We showed that images corrected with eddy and topup possessed higher FA values than images uncorrected and corrected with eddy_correct with trilinear (FSL default setting) or spline interpolation in most white matter skeletons, using both encoding schemes. Furthermore, the 60 directions encoding scheme was superior as measured by increased FA values to the 30 directions encoding scheme, despite comparable acquisition time. This study supports the combination of eddy and topup as a superior correction tool in diffusion imaging rather than the eddy_correct tool, especially with trilinear interpolation, using 60 directions encoding scheme.

## Introduction

Diffusion magnetic resonance imaging (MRI) is a unique and noninvasive tool to detect the white matter trajectory and integrity in vivo [1]. However, this technique suffers from spatial distortion and signal pileup or dropout, originating from local susceptibility-induced field gradient, since ultrafast acquisition techniques, such as echo-planar imaging (EPI), are exclusively employed to measure minute motion of water molecules in the brain tissue without motion-induced artifacts. Furthermore, eddy current resulting from the strong motion-probing gradients (MPG) is another source of geometric distortion, theoretically constrained by scale, shear, and translation deformations. To resolve these problems, several methods have been advocated. Multireference [2], field map with point-spread function mapping [3], and k-space traversal [4]–[6] can correct for susceptibility-induced distortions. Post-processing registration [7]–[12], twice-refocused spin echo [13] and characterization of the 3-D eddy current field with linear response theory [14] can be used to compensate for the eddy current induced distortion. Finally, k-space traversal combined with improvement of the method of Bowtell [15], [16] allows for both susceptibility and eddy current correction.

Although these methods are sometimes efficient to unwarp spatial distortion in diffusion imaging, several issues remain [6], [14], [15]: (a) subject motion during the acquisition of reference and diffusion-weighted images, resulting in misregistration between the two images and unwarping error; (b) prolonged scanning time to acquire reference images which do not contribute to functional or structural analyses of the brain; (c) inefficient correction of signal pileup or dropout; (d) signal intensity difference in areas of high signal intensity on non–diffusion-weighted images and low intensity on diffusion-weighted images (i.e., cerebrospinal fluid), and coherent white matter fascicles with high or low signal intensity, depending on MPG directionality, resulting in inefficient image registration and the erroneous calculation of diffusion properties; and (e) longer echo time, resulting in undesirable signal loss because of T2 decay. Furthermore, most proposed techniques can be applicable either to susceptibility or eddy-current induced distortion alone with a few exceptions [14], [15]. More importantly, subject motion and eddy current induced distortion cannot be separated with registration based techniques, which are compensatory operations. Therefore, the efficient distortion correction method in diffusion imaging remains to be an open question.

The Functional Magnetic Resonance Imaging of the Brain Software Library (FSL) offers a comprehensive toolset to analyze neuroimages for functional and structural connectivity, and morphometry (http://fsl.fmrib.ox.ac.uk/fsl/fslwiki) [17]. Initially, FSL provided a tool, named “eddy_correct”, to correct eddy current-induced image stretching, shearing, and translation on the basis of a classical affine transformation, but it did not offer the function to mitigate susceptibility-induced distortion and signal pileup. The “topup” and “eddy” FSL tools have been recently developed to estimate susceptibility and eddy current induced distortions, respectively, and correct them simultaneously [4], [18]–[20].

The calculation of diffusion tensor properties (i.e., fractional anisotropy; FA, and mean diffusivity) requires a minimum of 6 diffusion-weighted images with noncollinear MPG directions and 1 non–diffusion-weighted image. However, several diffusion-encoding schemes have been proposed for precise and robust measures, one of which is based on electrostatic repulsive forces. Among them, 30 and 60 noncollinear directions, with a ratio of the total number of diffusion-weighted images over non–diffusion-weighted images equal to 5 [21], [22], have been validated in clinical settings [23].

The present study compared the diffusion-derived FA values of non–distortion-corrected images (NC), images corrected with the eddy_correct tool (EC), and images corrected with the eddy and topup tools (ET), in a voxel-wise manner with Tract-Based Spatial Statistics (TBSS) [24]. Furthermore, the diffusion-weighted images were acquired with 2 acquisitions of 30 noncollinear MPG directions and 1 acquisition of 60 noncollinear MPG directions, with equivalent acquisition time. To our knowledge, this is the first report comparing uncorrected diffusion-weighted images to those corrected with the eddy_correct tool or a combination of the eddy and topup tools, using 30 and 60 directions encoding schemes in the human brain.

## Materials and Methods

### Participants

The subjects were 10 healthy volunteers (7 females; mean age: 34.0±5.5 years; range: 27–43 years). The ethical committee of the Nihon University Itabashi Hospital approved this study (No. RK121109-08). This was part of the ongoing research project investigating brain structural and functional alterations in patients with glossodynia. The exclusion criteria were drug use (antihypertensive, antianxiety, or antidepressive agents) or abuse, previous head trauma and operation, claustrophobia, diabetes, anemia, vitamin deficiency, and infections such as candidiasis. At least one of the trained neuroradiologists (O.A., T.S., or J.K.) evaluated all the anatomical MRI scans, including T1-weighted and T2-weighted images obtained in the same session, and found no gross abnormality in any of the participants. All subjects specifically consented to publication of medical information, including MR images, as well as participation in the study and written informed consents were obtained from them after a complete explanation of the study.

### MRI acquisition

The MRI data were obtained using a 1.5-T scanner (Achieva 1.5T; software version 3.2.1, Philips Medical Systems, The Netherlands) at the Nihon University Itabashi Hospital. Spin-echo EPI was used to obtain 60 contiguous axial images (repetition time/echo time = 8100/88.66 ms; spatial resolution = 2.5×2.5×2.5 mm) using an 8-channel phased-array head coil with a parallel imaging factor of 2. The MPG directions were conformed to 30 noncollinear directions on the basis of electrostatic repulsive forces (number of excitation = 5 for non–diffusion-weighted image; 1 for diffusion-weighted images), and to 60 noncollinear directions (number of excitation = 10 for non–diffusion-weighted image; 1 for diffusion-weighted images) with a b value of 1000 s/mm^2^ [21]. The ratio of the total number of diffusion-weighted over non–diffusion-weighted images was determined on the basis of previous literature [21]–[23]. The multiple non–diffusion-weighted images were averaged in-line to yield a single non–diffusion-weighted image from the scanner.

For the 30 directions encoding, 2 sets of diffusion images were acquired with the same parameters, except for the phase-encode blips which had opposing polarities along the anteroposterior direction. The acquisition time was 5 min 7 s per session, and the total scan time to encode 30 directions was 10 min 14 s. For the 60 directions encoding, only the non–diffusion-weighted images were acquired with 2 sets of phase encoding blips of opposite polarity (acquisition time: 3 min 14 s) and diffusion-weighted images were obtained with forward phase-encoding blips (acquisition time: 8 min 6 s). The total scan time for the 60 directions encoding protocol was 11 min 20 s, which was comparable with the 30 directions encoding protocol.

### Image processing

These digital imaging and communication in medicine (DICOM) images were transferred to a Linux workstation (HP Z820 workstation; Hewlett-Packard Japan, Tokyo) comprised of a CentOS 6.5 (64-bit version; 48 GBs memory) and dual central processing units (Intel Xeon Processor E5-2630 v2, Santa Clara, CA). The Sun Grid Engine grid computing cluster software system allowed parallel processing on this workstation. All DICOM images were converted into Neuroimaging Informatics Technology Initiative (NIfTI) format using the MRIcron tool named dcm2nii (http://www.mccauslandcenter.sc.edu/mricro/mricron/install.html). Then, the corresponding diffusion images, with opposing polarities of phase-encode blips for 30 and 60 directions encoding, were merged and treated as NC30 and NC60 images, respectively, both of which had 62 imaging volumes. Next the eddy current correction was applied with the eddy_correct tool [17] using the default settings, and these corrected images were processed at later stages as EC30 with trilinear interpolation for 30 directions, and as EC60 with trilinear interpolation for 60 directions encoding. In this step of the image processing, the first non–diffusion-weighted image was set as the target image, into which the remaining 61 volumes were registered. In the default setting of FSL, eddy_correct uses a trilinear function as an interpolation method and does not have the option selecting other interpolation methods. Therefore, we modified the script of eddy_correct and the interpolation function was changed from the default trilinear to spline, and the created images were EC30 and EC60 with spline interpolation. Finally, the topup and eddy tools were applied to the NC30 and NC60 images, which were named ET30 and ET60, respectively. Topup estimates the susceptibility-induced off-resonance field from pairs of images, with reversed phase-encode blips and distortions going in opposite directions (http://fsl.fmrib.ox.ac.uk/fsl/fslwiki/TOPUP) [4]. Two non–diffusion-weighted images with opposed phase encoding polarities were extracted, and susceptibility induced distortion was estimated with topup. The eddy tool corrects image distortions by assuming that diffusion signals obtained from 2 MPG directions with a small angle difference are similar, combining the correction for susceptibility and eddy currents/movements (http://fsl.fmrib.ox.ac.uk/fsl/fslwiki/EDDY). These diffusion images were processed with the FSL tool “dtifit,” and FA images were created for NC, EC, and ET. For the analyses with TBSS, FMRIB58_FA was set as the target image used in the registrations [24]. The value that thresholded the mean FA skeleton image was set at 0.2. All FA images were attached as supporting Information (Data S1).

### Statistical analyses

Permutation-based tests were conducted using the FSL tool “randomise_parallel,” with 50,000 permutations and the threshold-free cluster enhancement option [24]. Age and gender were not treated as covariates because their effects could be cancelled out in a paired analysis.

In the first step, paired *t*-tests were conducted between NC30 and EC30 with trilinear, and between NC60 and EC60 with trilinear interpolation. Second, tripled paired *t*-tests were conducted between NC30, EC30 with spline, and ET30, and between NC60, EC60 with spline interpolation, and ET60, separately. Third, a paired *t*-test was conducted between images derived from 30 and 60 directions encoding with the best performance, on the basis of the results of the tripled paired *t*-tests. We assumed that misregistration due to spatial distortions would result in lower FA values in tensor calculation. Although both eddy currents and subject movement can cause artificially elevated FA values at the interface between structures with high and low diffusivities, for example, gray matter and cerebrospinal fluid, the white matter skeleton is not the case. Therefore, the image with the best performance should have the highest FA values with TBSS analysis. The significance level was set at a *P* value of no more than 0.05 with family-wise error (FWE) correction for multiple comparisons.

## Results

### Visual inspections of the correction efficiency

**Figure 1:**
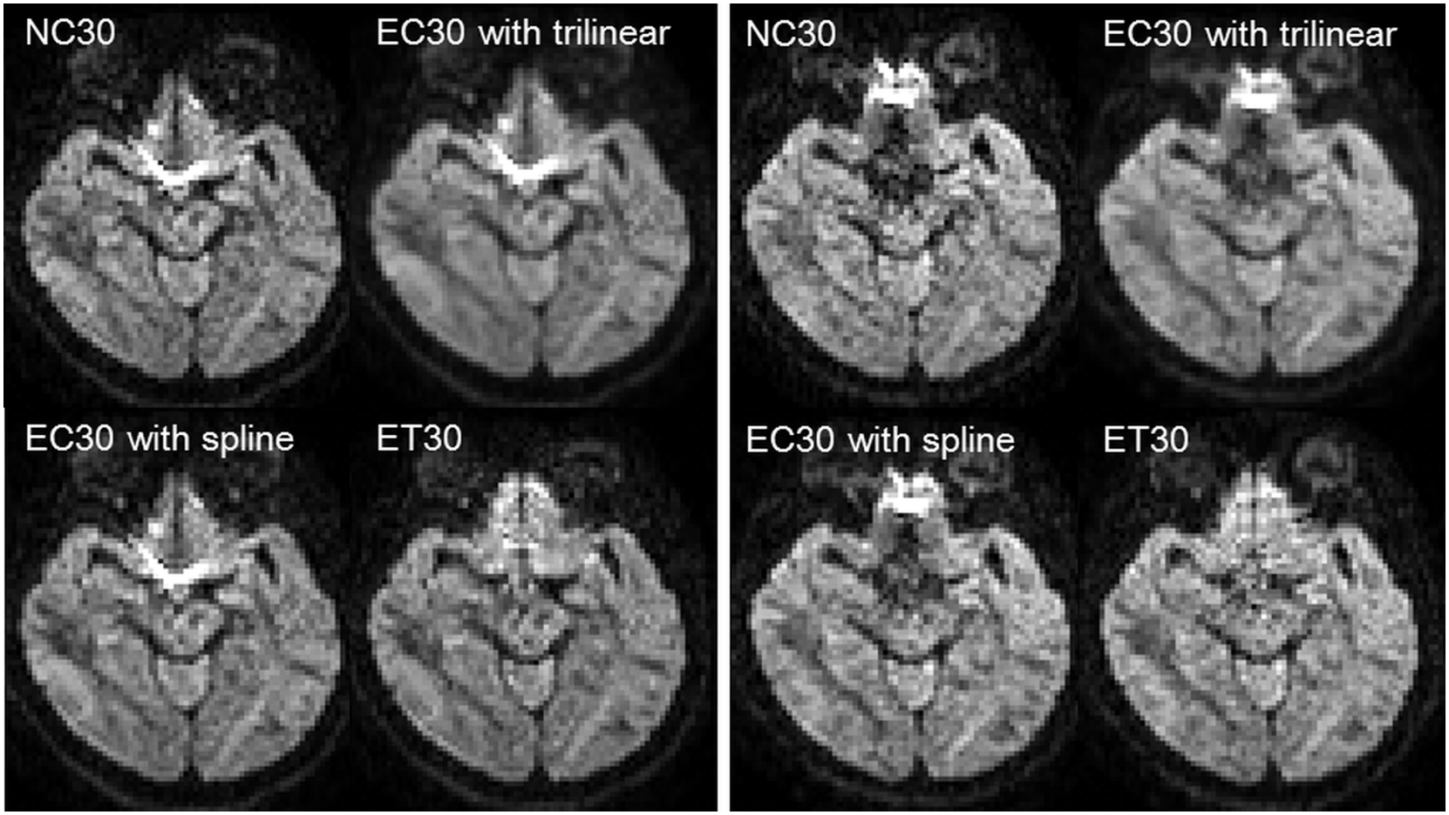
Representative diffusion-weighted images with the 30 directions encoding scheme. The images were from the same subject and slice location, with forward phase-encoding blips (left panel), and with reversed phase-encoding blips (right panel). NC30 had an artifactual signal pileup around the frontal base of the skull, which was corrected not in EC30 with trilinear or spline but in ET30. The EC30 with trilinear interpolation were blurred, compared with EC30 with spline interpolation.

Figure 1 showed representative diffusion-weighted images with the 30 directions encoding scheme. Diffusion images with forward and reverse phase encode blips were shown on the left and right panel, respectively. Before correction, NC30 showed an artifactual signal pileup around the frontal base of the skull with both forward and reversed phase encoding blips. These artifacts were not corrected in EC30 with trilinear or spline interpolation, but were corrected in ET30. With respect to EC30, images blurring occurred with trilinear interpolation (FSL default setting), but not with spline interpolation. Representative movie files of the sagittal, coronal, and axial non–diffusion-weighted and diffusion-weighted images were shown in Fig. S1–S4. Compared with those of NC (Fig. S1), EC with trilinear (Fig. S2) and spline interpolation (Fig. S3), brain surfaces were well-registered among ET images (Fig. S4) by visual inspection. Furthermore, concave or convex distortions were alleviated in ET images.

**Figure 2:**
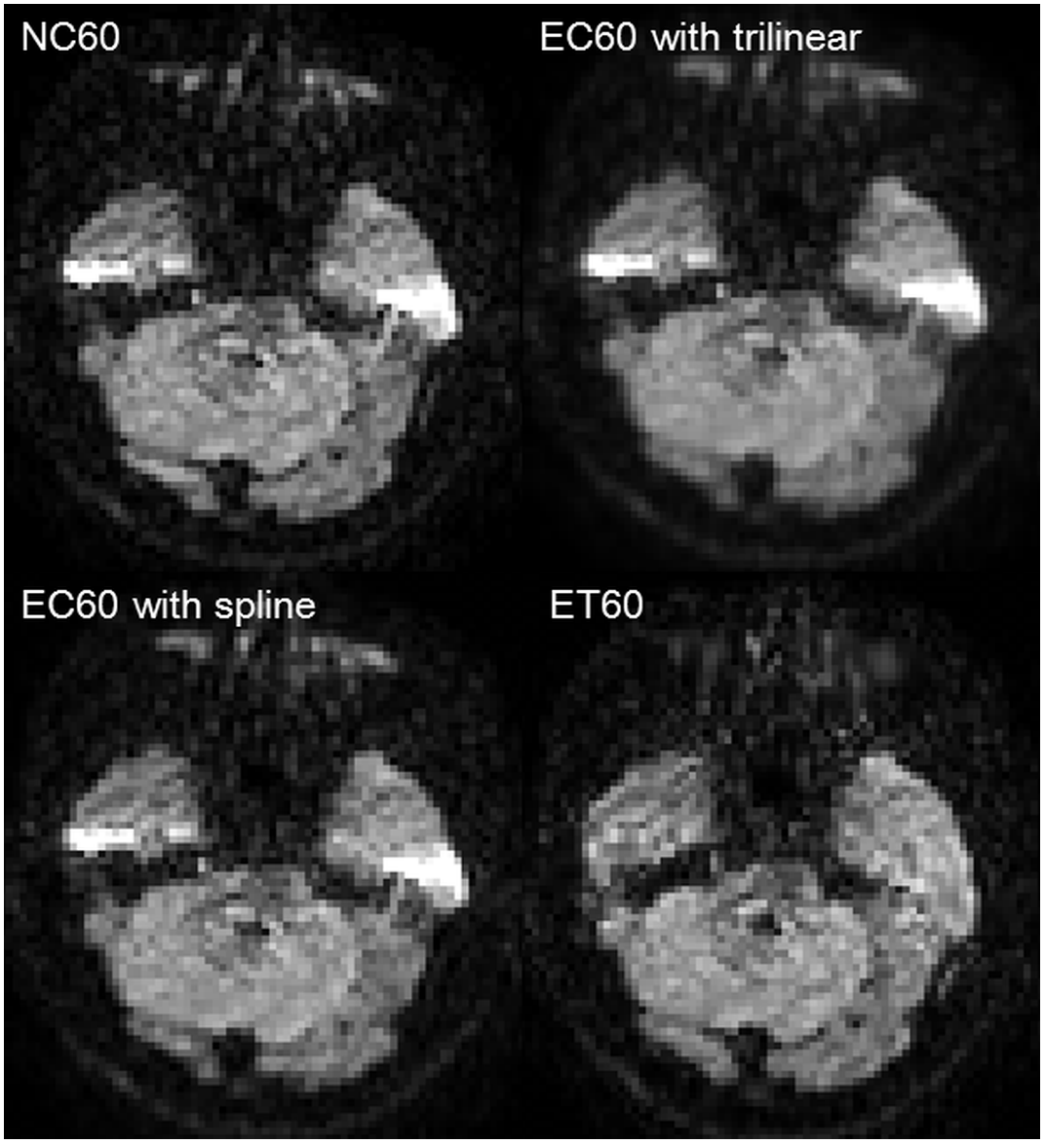
Representative diffusion-weighted images with the 60 directions encoding scheme. The images were from the same subject and slice location. The NC60 with forward phase-encoding blips had an artifactual signal pileup around the temporal base of the skull, which was not corrected in EC60, but corrected in ET60. Again, the EC30 with trilinear were blurred, compared with EC30 with spline interpolation.

Figure 2 showed representative diffusion-weighted images acquired with the 60 directions encoding and forward phase encoding blips. Before correction, NC60 showed an artifactual signal pileup around the temporal base of the skull, which was not corrected in EC60, but corrected in ET60. Likewise, image blurring was only detected in EC60 with trilinear interpolation (FSL default setting). Therefore, both acquisition schemes offered better images with the eddy and topup corrections. Representative movie files of the sagittal, coronal, and axial non–diffusion-weighted and diffusion-weighted images were shown in Fig. S5–S8. Compared with those of NC (Fig. S5), EC with trilinear (Fig. S6) and spline interpolation (Fig. S7), brain surfaces were well-registered among ET images (Fig. S8) by visual inspection. Furthermore, concave or convex distortions were alleviated in ET images.

### Comparisons between NC and EC images with trilinear interpolation

**Figure 3:**
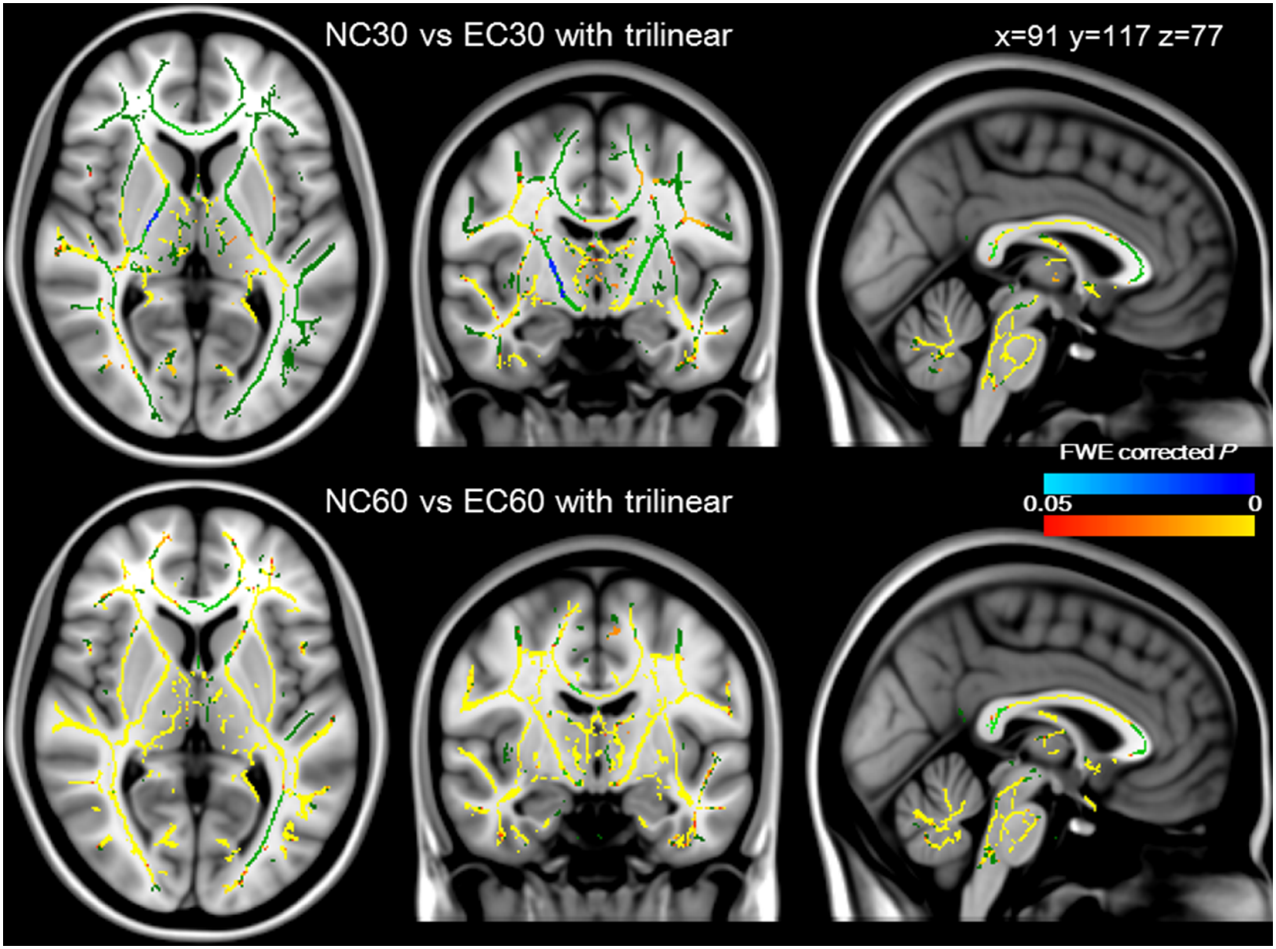
Comparisons between NC and EC images with trilinear interpolation. The white matter skeletons with the higher FA for NC30 or NC60 were shown in red/yellow, and those with higher FA for EC30 or EC60 with trilinear interpolation were shown in blue/lightblue. The upper and lower row showed paired comparisons between NC30 and EC30 with trilinear, NC60 and NC60 with trilinear interpolation, respectively. NC30 and NC60 had higher FA values in most white matter skeletons, compared with EC30 and EC60 with trilinear interpolation, except in the posterior limb of the right internal capsule in the upper row. These data were overlaid onto the MNI152_T1_1 mm template, with the mean FA skeletons shown in green. The significance level was set at a *P* value of <0.05 with FWE correction.

Paired *t*-tests with TBSS were conducted between NC30 and EC30 with trilinear interpolation, and between NC60 and EC60 with trilinear interpolation (Fig. 3). The white matter skeleton with significantly higher FA for NC30 or NC60 were shown in red/yellow, and those with the significantly higher FA for EC30 or EC60 were shown in blue/light blue (FWE corrected *P*<0.05 for all comparisons). Surprisingly, most of the white matter skeleton showed higher FA values for NC30 and NC60 than those for EC30 and EC60 with trilinear interpolation. The only exception was in the posterior limb of the right internal capsule between NC30 and EC30 with trilinear interpolation. Therefore, we decided not to conduct further analysis of EC30 or EC60 with trilinear interpolation (FSL default setting).

### Efficiency of correction schemes acquired with 30 directions diffusion encoding

**Figure 4:**
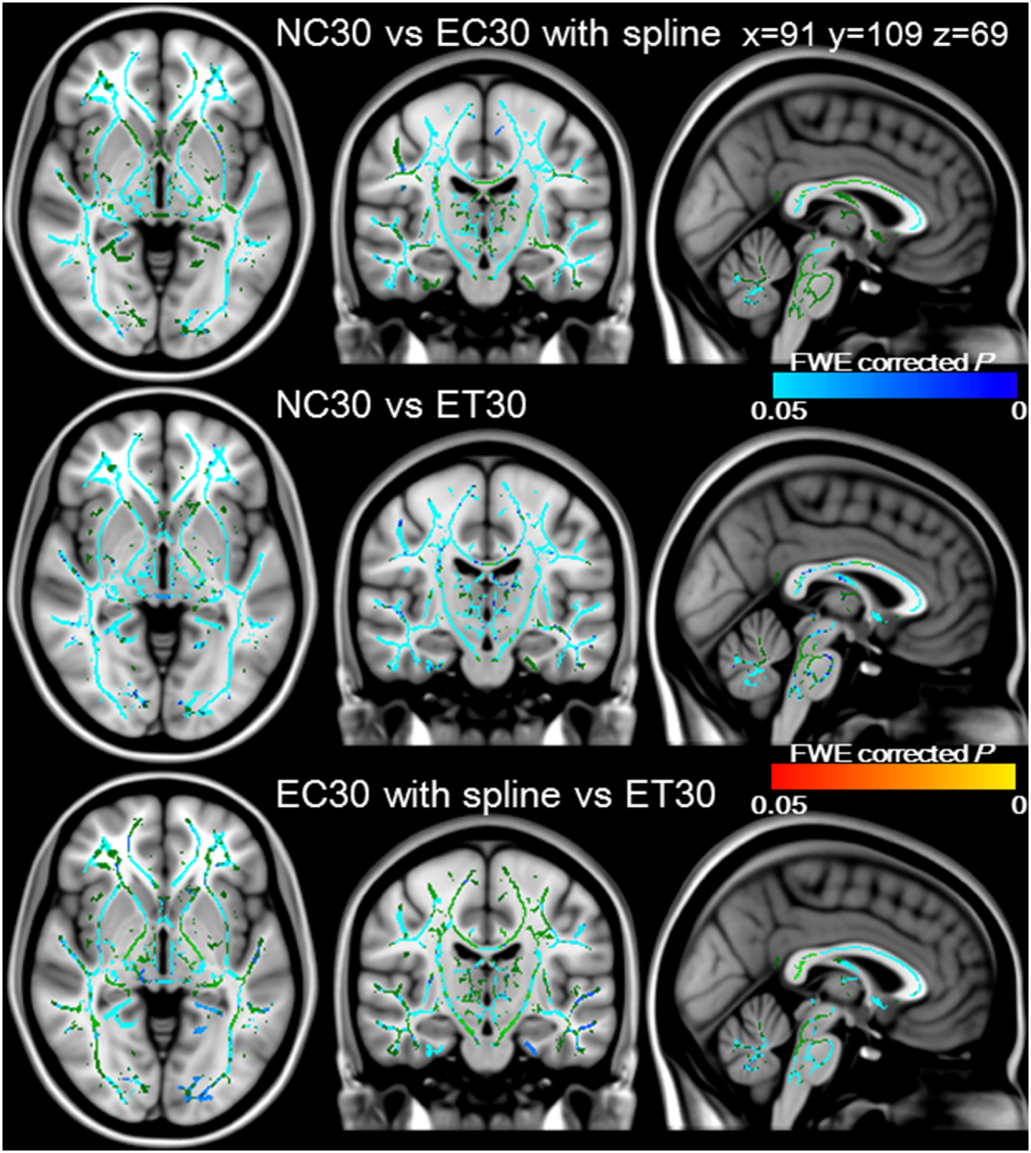
Efficiency of correction schemes acquired with 30 directions diffusion encoding. Tripled paired group comparisons with TBSS were conducted between NC30 and EC30 with spline interpolation in the upper, NC30 and ET30 in the middle, and EC30 with spline interpolation and ET30 in the lower row. The FA values for ET30 were significantly higher than those for NC30 or EC30 in most white matter skeletons (blue/lightblue). The FA values for EC30 with spline interpolation were significantly higher than those for NC30 (blue/lightblue), and lower than those for ET30 in most white matter skeletons. These data were overlaid onto the MNI152_T1_1 mm template, with the mean FA skeletons shown in green. The significance level was set at a *P* value of <0.05 with FWE correction.

Figure 4 showed tripled paired *t*-tests with TBSS between NC30 and EC30 with spline interpolation in the upper, between NC30 and ET30 in the middle, and between EC30 with spline interpolation and ET30 in the lower row, respectively. The FA values for ET30 were significantly higher than those for NC30 or EC30 with spline in most of the white matter skeleton (blue/light blue), and there was no skeleton where FA values for ET30 was significantly lower than those for the others (FWE corrected *P*<0.05 for all comparisons). In addition, the FA values for NC30 were never significantly higher than those for the others in the entire skeleton. Finally, the FA values for EC30 with spline interpolation were significantly higher than those for NC30 (blue/light blue), and lower than those for ET30 (red/yellow) in most of the white matter skeleton (FWE corrected *P*<0.05 for all comparisons). These data suggested that ET30 provided the most efficient image correction.

### Efficiency of correction schemes acquired with 60 directions diffusion encoding

**Figure 5:**
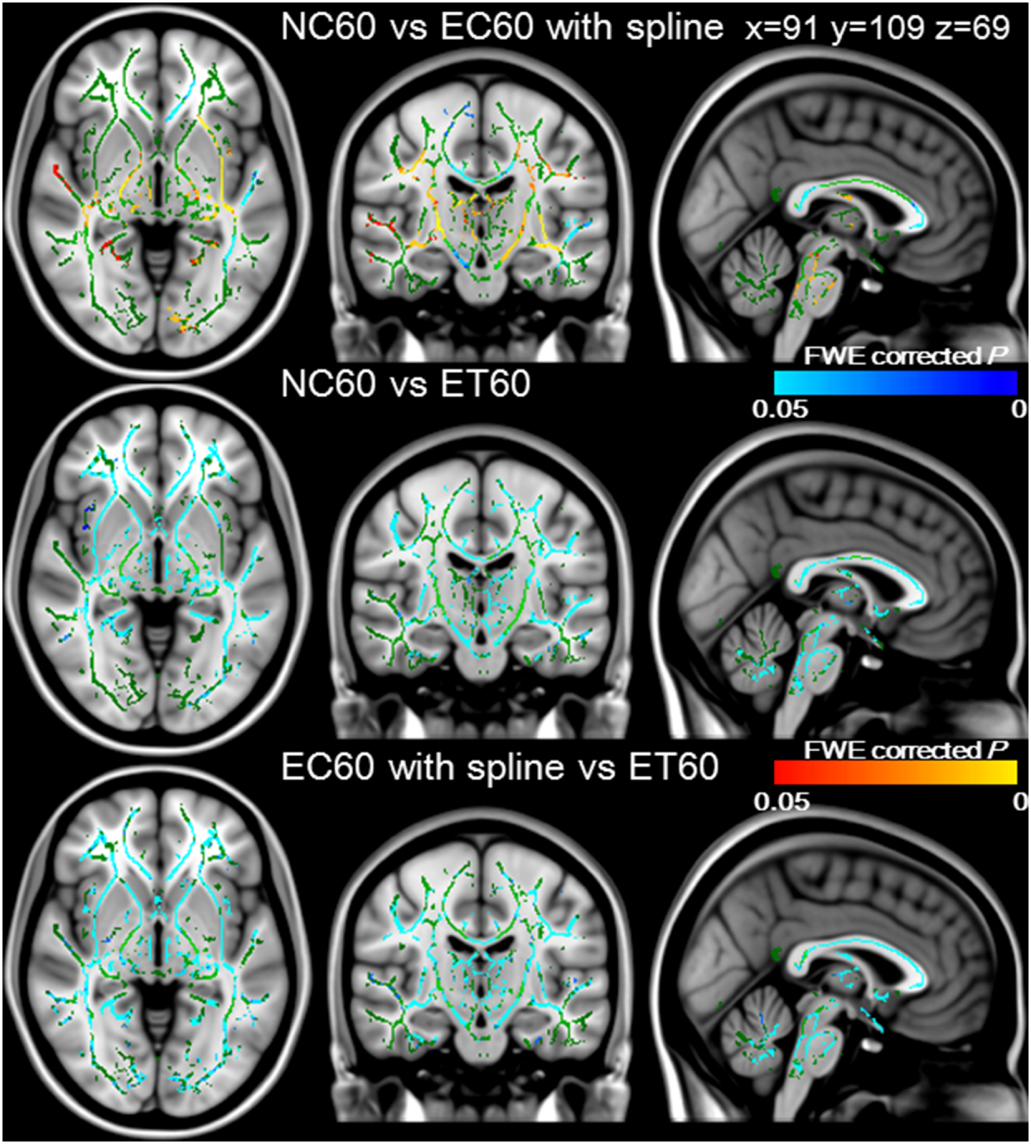
Efficiency of correction schemes acquired with 60 directions diffusion encoding. Tripled paired group comparisons with TBSS were conducted between NC60 and EC60 with spline interpolation in the upper, NC60 and ET60 in the middle, and EC60 with spline and ET60 in the lower row. The FA values for ET60 were significantly higher than those for NC60 or EC60 in most of the white matter skeleton (blue/lightblue). In contrast, white matter skeleton with significantly higher (blue/lightblue) and lower (red/yellow) FA values for EC60 with spline interpolation than those for NC60 were observed in various areas. These data were overlaid onto the MNI152_T1 template, and the mean FA skeleton is shown in green. The significance level was set at a *P* value of <0.05 with FWE correction.

Figure 5 showed tripled paired *t*-tests with TBSS between NC60 and EC60 with spline interpolation in the upper, between NC60 and ET60 in the middle, and between EC60 with spline interpolation and ET60 in the lower row, respectively. As for the previous scheme, the FA values for ET60 were significantly higher than those for NC60 or EC60 with spline interpolation in most of the white matter skeleton, and there was no skeleton where FA values for ET60 was significantly lower than those for the others (FWE corrected *P*<0.05 for all comparisons). In contrast, various areas of the white matter skeleton showed significant higher (blue/light blue) or lower (red/yellow) FA values for EC60 with spline interpolation than those for NC60 (FWE corrected *P*<0.05 for all comparisons). Therefore, ET60 exhibited the most efficient image correction.

### Comparison of ET30 and ET60 images

**Figure 6:**
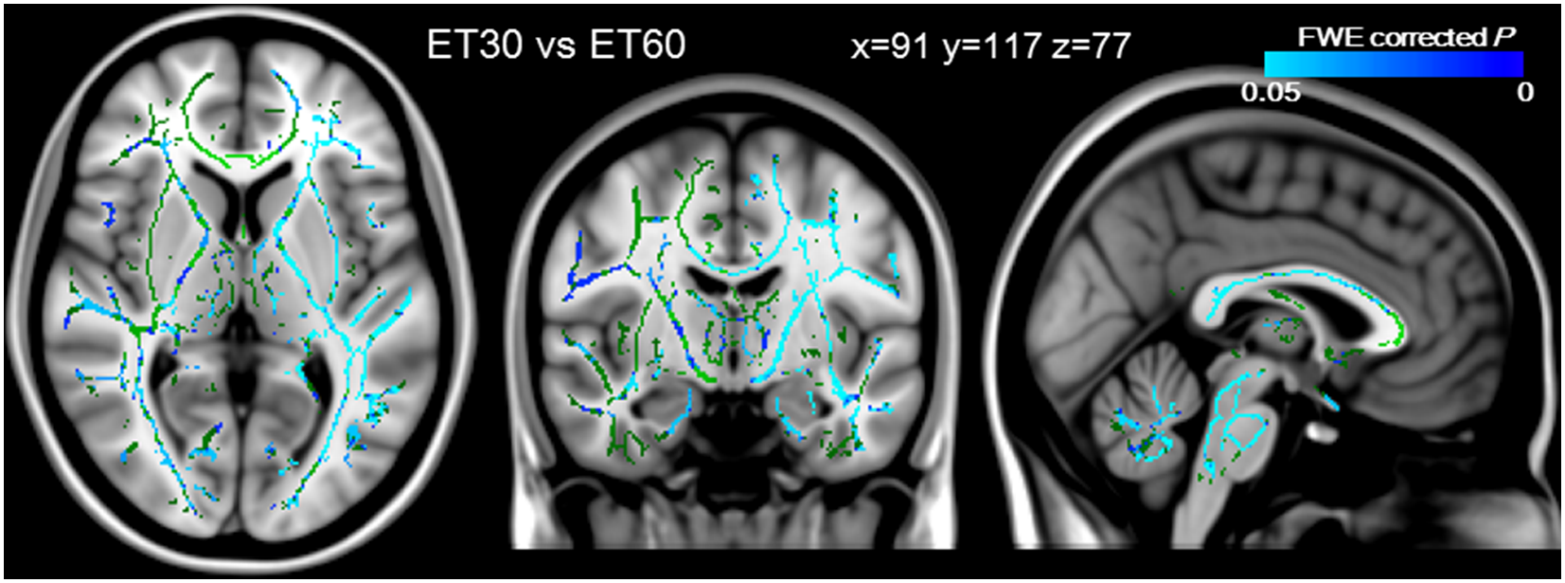
Comparison of ET30 and ET60 images. The FA values for ET60 were significantly higher than those for ET30 in most of the white matter, with slight left hemisphere predominance. These data were overlaid onto the MNI152_T1 template, and the mean FA skeleton is shown in green. The significance level was set at a *P* value of <0.05 with FWE correction.

The two most efficient correction protocols were compared by paired *t*-test with TBSS (Fig. 6). The FA values for ET60 were significantly higher than those for ET30 in widespread white matter, with slight left hemisphere predominance (blue/lightblue; FWE corrected *P*<0.05). On the other hand, there was no skeleton where FA value for ET60 was significantly lower than that for ET30. Altogether, these data suggested that the 60 directions encoding scheme provided more reliable FA measurements than the 30 directions encoding scheme after the eddy and topup corrections.

## Discussion

MRI remains the most powerful noninvasive neuroimaging technique to clarify task-related cortical activation, functional connectivity at rest, cerebral perfusion, as well as regional morphology and metabolic activity. Among the recent applications, diffusion imaging can reveal structural connectivity and white matter integrity. However, structural distortion is a serious problem for these methods since EPI-based acquisition is employed. In terms of diffusion imaging, two major factors contribute to spatial distortion: local susceptibility gradient and eddy current due to the strong MPG application. Without distortion correction, calculations of diffusion properties may be erroneous because of misregistration.

The present study compared the FA values of uncorrected images with those of images corrected with the eddy_correct tool or a combination of the topup and eddy tools. The latter exhibited the highest FA values in most of the white matter skeleton with the 30 directions as well as 60 directions encoding schemes. Furthermore, the FA values of images corrected with the topup and eddy tools were significantly higher in widespread white matter with 60 directions than with 30 directions. Slight residual misregistrations between the two images, especially diffusion-weighted images with opposing phase-encoding directions in the ET30 set, might remain, resulting in the observed reduction in FA. In the current study, however, we did not acquire two imaging sets with the phase encoding blips of the same polarity in terms of 30 directions diffusion encoding, and could not compare them with 60 directions encoded images. In contrast, residual misregistrations between the two images, especially diffusion-weighted images with opposing phase-encoding directions in the ET30 set, can cause artificially elevated FA values at the interface between structures with high and low diffusivities, for example, gray matter and cerebrospinal fluid. Furthermore, at the resolution being probed in this study misregistation of the white matter tracts would have a similar poor correspondence with the gray/white matter boundary and result in abnormally high FA measures on the edge of the white matter tracts. In ET60, however, there was only one data set of diffusion-weighted images. Misregistration between two sets of image data were not applicable and was not a reason for higher FA values in ET60, which might exert a minor effect on the results. Although the exact cause remains unknown, the 60 directions encoding scheme is believed to be superior as measured by increased FA values to the 30 directions encoding scheme because there was no white matter skeleton with FA values for 30 directions higher than those for 60 directions in the entire hemispheres. Furthermore, a higher number of encoding directions increases angular resolution, which might facilitate the analysis of complicated diffusion parameters beyond diffusion tensor, such as high angular resolution diffusion imaging [25]. Previous reports investigating the optimal number of MPG suggested that about or at least 30 directions offered the best performance. However, one report did not evaluate 60 directions [23], and the other study only used a simulation model [21]. Future imaging studies, however, are clearly needed to evaluate the difference between FA values for 30 and 60 directions encoding scheme with the phase encoding blips of the same polarity.

The topup tool uses the reversed direction of phase encoding to estimate the EPI distortion caused by local susceptibility gradient [4]. The estimated distortion field is then advanced into a Gaussian process predictor that uses all the data to estimate the eddy current-induced field inhomogeneities and head motion for each imaging volume [18]. All these distortions are corrected in a single process using the eddy tool [18]–[20]. In contrast, the eddy_correct tool can register diffusion-weighted images into the reference image, usually a non–diffusion-weighted image which has a different contrast from diffusion-weighted images, with a classical affine transformation-based method. But this tool cannot correct susceptibility-induced distortion, which might explain why images corrected with the eddy_correct tool had significantly lower FA values than those corrected with the topup and eddy tools. Sotiropoulos et al. found that, with measurements of sum of squared differences in signal intensities within the brain, an affine-based correction provided better results than without correction, but clearly performed worse than the Gaussian Process approach, which achieves a better registration between volumes [20]. This study was in good agreement with our current results. Furthermore, the FA values for EC60 even with spline interpolation were significantly lower than those for uncorrected images in some areas of the white matter skeleton. In addition, the FA values for EC30 and EC60 with trilinear interpolation, the default interpolation methods in the eddy_correct tool, were significantly lower than those for NC30 and NC60 in widespread white matter. Therefore, we strongly recommended the topup and eddy tools for distortion correction in diffusion imaging, rather than the eddy_correct tool, especially with the default trilinear interpolation.

The correction method using topup and eddy tools has a few limitations. First, when subjects move between two acquisitions, with different phase encoding polarities, the corrected images would be different from the real shape of the brain because topup attempts to estimate the distortion correction field that will maximize the similarities between the corrected images. If significant movement occurs, another acquisition will be needed. On the other hand, most image correction methods share this problem. Second, we did not apply MPG reorientation when unwarping images. When a brain is transformed onto another one, such as the standard brain (i.e. spatially normalized template), which is quite different from a subject brain, MPG reorientations would be indispensable according to the rotation matrix around the x, y, and z axes. However, in the current settings, distortion-corrected images have the native shape of the brain, and MPG has been applied to the native shape. Therefore, we believe that MPG reorientation was unnecessary. Finally, we did not use over 60 gradient directions because the amount of acquisition time for diffusion imaging was limited in clinical or even research settings. Because this study investigated brain alterations in subjects diagnosed with glossodynia, other imaging sequences might be required to evaluate functional connectivity and structural deficit. The effect of a hundred or more MPG directions on diffusion imaging is a subject for future studies, especially after multiband technology becomes routinely feasible [19], [20].

In conclusion, structural distortions cause misregistration in non–diffusion-weighted and diffusion-weighted images during tensor calculation, resulting in lower FA values. The present study showed that ET images had higher FA values than EC images, which suggested that distortion correction with the topup and eddy tools might be indispensable for accurate measurements of diffusion parameters. Furthermore, the 60 directions encoding scheme was superior as measured by increased FA values to the 30 directions encoding scheme based on electrostatic repulsive forces, despite comparable acquisition time.

## Supporting Information

Figure S1 **Representative movie files of non–diffusion-weighted and diffusion-weighted images of NC for the 30 directions encoding.** Sagittal, coronal, and axial non–diffusion-weighted and diffusion-weighted images of NC were shown.
(GIF)

Figure S2 **Representative movie files of non–diffusion-weighted and diffusion-weighted images of EC with trilinear interpolation for the 30 directions encoding.** Sagittal, coronal, and axial non–diffusion-weighted and diffusion-weighted images of EC with trilinear interpolation were shown.
(GIF)

Figure S3 **Representative movie files of non–diffusion-weighted and diffusion-weighted images of EC with spline interpolation for the 30 directions encoding.** Sagittal, coronal, and axial non–diffusion-weighted and diffusion-weighted images of EC with spline interpolation were shown.
(GIF)

Figure S4 **Representative movie files of non–diffusion-weighted and diffusion-weighted images of ET for the 30 directions encoding.** Sagittal, coronal, and axial non–diffusion-weighted and diffusion-weighted images of ET were shown. Compared with those of NC (Fig. S1), EC with trilinear (Fig. S2) and spline interpolation (Fig. S3), brain surfaces were well-registered among ET images (Fig. S4) by visual inspection. Furthermore, concave or convex distortions were alleviated in ET images.
(GIF)

Figure S5 **Representative movie files of non–diffusion-weighted and diffusion-weighted images of NC for the 60 directions encoding.** Sagittal, coronal, and axial non–diffusion-weighted and diffusion-weighted images of NC were shown.
(GIF)

Figure S6 **Representative movie files of non–diffusion-weighted and diffusion-weighted images of EC with trilinear interpolation for the 60 directions encoding.** Sagittal, coronal, and axial non–diffusion-weighted and diffusion-weighted images of EC with trilinear interpolation were shown.
(GIF)

Figure S7 **Representative movie files of non–diffusion-weighted and diffusion-weighted images of EC with spline interpolation for the 60 directions encoding.** Sagittal, coronal, and axial non–diffusion-weighted and diffusion-weighted images of EC with spline interpolation were shown.
(GIF)

Figure S8 **Representative movie files of non–diffusion-weighted and diffusion-weighted images of ET for the 60 directions encoding.** Sagittal, coronal, and axial non–diffusion-weighted and diffusion-weighted images of ET were shown. Compared with those of NC (Fig. S5), EC with trilinear (Fig. S6) and spline interpolation (Fig. S7), brain surfaces were well-registered among ET images (Fig. S8) by visual inspection. Furthermore, concave or convex distortions were alleviated in ET images.
(GIF)

Data S1 **Preprocessed fractional anisotropy maps for all subjects.** The prefixes NC, EC, and ET indicate reconstructed images with no correction, eddy_correct with spline interpolation, and topup and eddy, respectively. NC30, EC30, and ET30 images were obtained from 30 directions diffusion encoding, and NC60, EC60, and ET60 from 60 directions encoding. The postfix for each filename denote subject number.
(GZ) Click here for additional data file.
