# Supplementary figures and images for: Efficacy of Distortion Correction on Diffusion Imaging: Comparison of FSL Eddy and Eddy_Correct Using 30 and 60 Directions Diffusion Encoding
Source: PLoS One. 2014 Nov 18;9(11):e112411. doi: 10.1371/journal.pone.0112411 (PMC4236106; doi:10.1371/journal.pone.0112411)

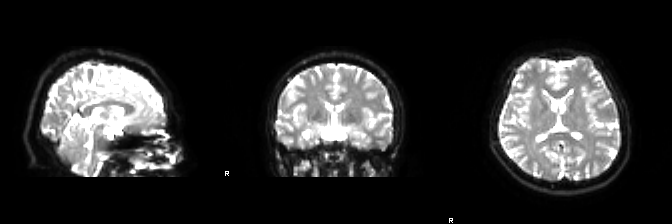

Supplement: Figure S1 — Representative movie files of non–diffusion-weighted and diffusion-weighted images of NC for the 30 directions encoding. Sagittal, coronal, and axial non–diffusion-weighted and diffusion-weighted images of NC were shown. (GIF) [file pone.0112411.s001.gif]

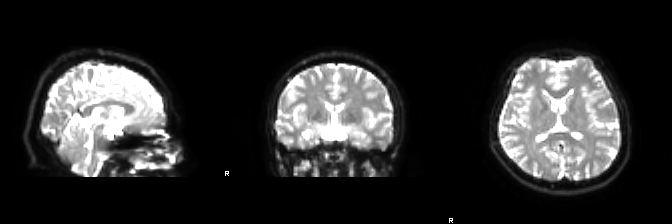

Supplement: Figure S2 — Representative movie files of non–diffusion-weighted and diffusion-weighted images of EC with trilinear interpolation for the 30 directions encoding. Sagittal, coronal, and axial non–diffusion-weighted and diffusion-weighted images of EC with trilinear interpolation were shown. (GIF) [file pone.0112411.s002.gif]

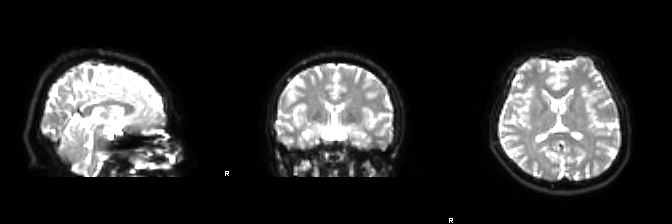

Supplement: Figure S3 — Representative movie files of non–diffusion-weighted and diffusion-weighted images of EC with spline interpolation for the 30 directions encoding. Sagittal, coronal, and axial non–diffusion-weighted and diffusion-weighted images of EC with spline interpolation were shown. (GIF) [file pone.0112411.s003.gif]

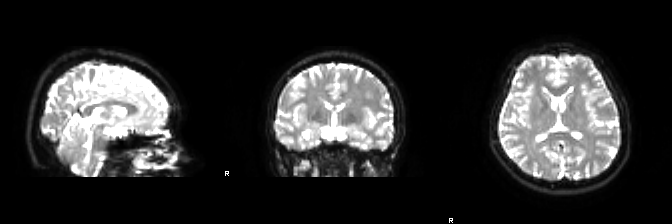

Supplement: Figure S4 — Representative movie files of non–diffusion-weighted and diffusion-weighted images of ET for the 30 directions encoding. Sagittal, coronal, and axial non–diffusion-weighted and diffusion-weighted images of ET were shown. Compared with those of NC (Fig. S1), EC with trilinear (Fig. S2) and spline interpolation (Fig. S3), brain surfaces were well-registered among ET images (Fig. S4) by visual inspection. Furthermore, concave or convex distortions were alleviated in ET images. (GIF) [file pone.0112411.s004.gif]

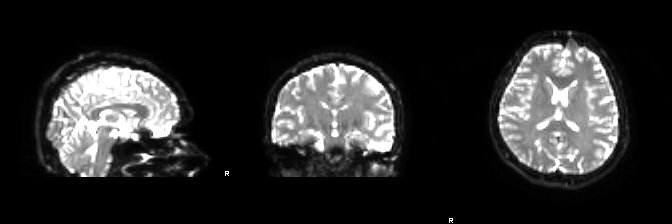

Supplement: Figure S5 — Representative movie files of non–diffusion-weighted and diffusion-weighted images of NC for the 60 directions encoding. Sagittal, coronal, and axial non–diffusion-weighted and diffusion-weighted images of NC were shown. (GIF) [file pone.0112411.s005.gif]

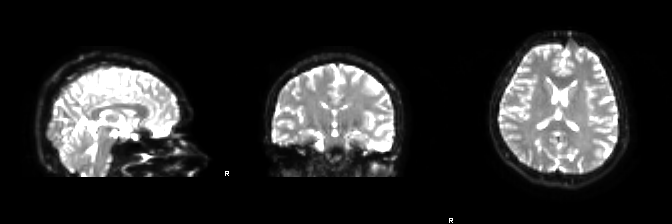

Supplement: Figure S6 — Representative movie files of non–diffusion-weighted and diffusion-weighted images of EC with trilinear interpolation for the 60 directions encoding. Sagittal, coronal, and axial non–diffusion-weighted and diffusion-weighted images of EC with trilinear interpolation were shown. (GIF) [file pone.0112411.s006.gif]

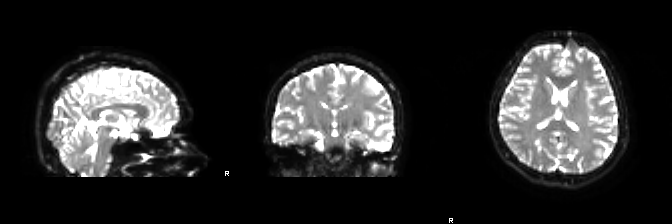

Supplement: Figure S7 — Representative movie files of non–diffusion-weighted and diffusion-weighted images of EC with spline interpolation for the 60 directions encoding. Sagittal, coronal, and axial non–diffusion-weighted and diffusion-weighted images of EC with spline interpolation were shown. (GIF) [file pone.0112411.s007.gif]

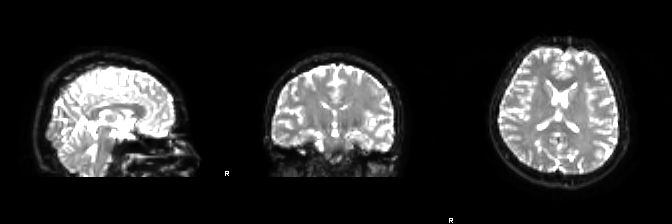

Supplement: Figure S8 — Representative movie files of non–diffusion-weighted and diffusion-weighted images of ET for the 60 directions encoding. Sagittal, coronal, and axial non–diffusion-weighted and diffusion-weighted images of ET were shown. Compared with those of NC (Fig. S5), EC with trilinear (Fig. S6) and spline interpolation (Fig. S7), brain surfaces were well-registered among ET images (Fig. S8) by visual inspection. Furthermore, concave or convex distortions were alleviated in ET images. (GIF) [file pone.0112411.s008.gif]
